# Practical and Durable Flexible Strain Sensors Based on Conductive Carbon Black and Silicone Blends for Large Scale Motion Monitoring Applications

**DOI:** 10.3390/s19204553

**Published:** 2019-10-19

**Authors:** Yun Xia, Qi Zhang, Xue E. Wu, Tim V. Kirk, Xiao Dong Chen

**Affiliations:** 1 School of Chemical and Environmental Engineering, College of Chemistry, Chemical Engineering and Material Science, Soochow University, Suzhou Industrial Park Campus, Suzhou 215123, China; 20154009042@stu.suda.edu.cn (Y.X.); xdchen@mail.suda.edu.cn (X.D.C.); 2 Department of Chemical and Biochemical Engineering, Xiamen University, Xiamen 361005, China; 20620140153872@stu.xmu.edu.cn

**Keywords:** flexible, capacitive sensor, strain sensor, durable, wearable, low cost

## Abstract

Presented is a flexible capacitive strain sensor, based on the low cost materials silicone (PDMS) and carbon black (CB), that was fabricated by casting and curing of successive silicone layers—a central PDMS dielectric layer bounded by PDMS/CB blend electrodes and packaged by exterior PDMS films. It was effectively characterized for large flexion-angle motion wearable applications, with strain sensing properties assessed over large strains (50%) and variations in temperature and humidity. Additionally, suitability for monitoring large tissue deformation was established by integration with an in vitro digestive model. The capacitive gauge factor was approximately constant at 0.86 over these conditions for the linear strain range (3 to 47%). Durability was established from consistent relative capacitance changes over 10,000 strain cycles, with varying strain frequency and elongation up to 50%. Wearability and high flexion angle human motion detection were demonstrated by integration with an elbow band, with clear detection of motion ranges up 90°. The device’s simple structure and fabrication method, low-cost materials and robust performance, offer promise for expanding the availability of wearable sensor systems.

## 1. Introduction

With the development of miniaturized and portable computing devices and sensors and burgeoning interest in personalized healthcare and new concepts in human–machine interaction, smart wearable devices [1,2,3] have attracted wide interest because of their potential in health monitoring [4,5], motion tracking [6,7,8,9,10,11] and assessment of in vitro models of gastric motility [12,13]. Among these devices, “electronic skin” [14,15,16,17] has developed as a prominent class. Beginning in the 1970s, the concept emerged in science fiction, and in the new century, with the improvement of science and technology, electronic skin began to move from fiction to reality [18]. 

Early electronic skin consisted primarily of non-conductive elastomers and flexible conductive sensing elements. Their sensing mechanisms were also relatively simple and were generally divided into two types: Resistive [6,19] and capacitive [20,21,22,23]. In recent years, many researchers have pursued development of capacitive electronic skin, with carbon nanotubes (CNTs) [6,20,23,24,25], graphene [26,27,28] and carbon black [29,30] often used as conductive elements, while others have used silver nanowires (AgNW) [21,22,31] or metallic nanoparticles [32,33,34]. Silicones [35], spun elastic fibers [36] and conventional textiles [37] have been used as dielectric layers. Among them, the silicone poly(dimethyl siloxane) (PDMS) [35] is most often used as an inexpensive non-conductive elastomer, with its relative ease of fabrication and chemical modification and its low glass transition temperature contributing to its high flexibility [38]. All these systems have depended upon attachment of the conductive material to the surface of the flexible substrate, by spraying or other deposition methods [6,24,32,33,34,39,40,41,42,43,44,45]. Sensitive devices have been produced, but challenges remain, such as durability. The interface between the flexible substrate and its conductive coating must maintain its integrity, which can be challenging due to the risk of delamination and shedding [46,47]. Some researchers have reported endurance testing for durability [40,48], though typically over short durations. Other researchers have employed elastomeric ionic hydrogels or ionomer polymers as their conductive medium [49,50], pursuing long-term stability and biocompatibility. Ionic sensors [49,50] do not suffer from low-motion artifacts and have high sensitivity to external mechanical stimulation, but their production processes are relatively complex and may not be suitable for mass production. Therefore, it is still challenging to develop a flexible, conductive sensor material that is highly durable, offers simple assembly, has sufficient performance, is suitable for large deformation and flexion angle human motion and yet is low cost.

The conductive mechanism of carbon black [30] (CB) is explained by seepage theory [51,52], where as long as the distances between the carbon particles are small enough, conduction can be achieved. Guo et al. [29] developed a multilayer (10) textile-attached capacitive pressure sensor that used a PDMS/CB blend as its dielectric layer, with PDMS as shielding layers and organo-silicone adhesive as electrode layers. Tsouti et al. [30] suggested that it was possible to develop a simpler arrangement for a PDMS/CB based capacitive strain sensor, where a PDMS/CB blend was used for the electrodes and PDMS as the dielectric. However, their experimental work was limited to very low strain (0.05%) measurement of a cantilever. In 2018, during the development of this current work, Shintake et al. [53] published a characterization of a similar five layer arrangement (Ecoflex™ silicone dielectric layer sandwiched by blended silicone/CB conductive layers and packaged in silicone layers) over large scale deformation (500% strain) and over high numbers of repetitive strain cycles (10,100). This study also included sensor integration with a glove and monitoring of finger bending over ~20% sensor strain. 

In this paper we demonstrate how similar materials, in a simple five layer arrangement, can be made effectively at first hand and can be used in a large-strain (50%), durable, low-cost sensor that is suitable for use as a wearable device for monitoring high flexion-angle body motion and large tissue deformation. The device was prepared by sequential casting and curing of the silicone layers—a simple, low-cost, industrially relevant method. Durability during large strain (50%) repetitive motion was established via testing over 10,000 tensile strain cycles, while body motion monitoring was demonstrated by fixing planar sensors to paper elbow and knee bands, with tissue deformation examined via fixing a sensor to the silicone stomach of an in vitro model of human digestion. [54] The latter, by monitoring the peristaltic contractions that drive gastric motility, opens the possibility of enhancing in-demand in vitro models of gastric digestion [12,13]. Additionally, the effects of temperature and humidity upon sensor performance were examined.

## 2. Materials and Methods

### 2.1. Materials

Cabot XC-72R CB powder was purchased from Cabot Corporation (Boston, MA, USA); Sylgard-184 PDMS (silicone elastomer base and silicone elastomer curing agent) from Dow corning company (Midland, MI, USA); polyethylene terephthalate (PET) 0.05 and 0.2 mm thickness and Isopropanol from Sinopharm company (Shanghai, China); and poly(acrylic acid) (PAA) 50% solution from Aladdin (Shanghai, China).

The automatic film applicator (AFA-Ⅱ) was purchased from Shanghai Moderner company (Shanghai, China); and a height adjustable stopper (HAS) (1806B) from BEVS company (Shanghai, China).

### 2.2. Methods

#### 2.2.1. Fabrication

The device was fabricated from 3 PDMS layers and 2 PDMS/CB blend conductive layers, in an alternating configuration. The layers were bonded together by successively placing uncured layers on top of the cured stack and subsequently curing in an oven. Further details follow.

(1) Production of PAA coated PET film

Thirty-six g isopropanol was added to 4 g PAA 50% solution [55] and the solution was stirred for 5 min to obtain a white suspension. This solution was then sprayed onto a 0.05 mm thick PET film to ensure the sensor layer was easily removed from the PET mold.

(2) Flexible conductive silicone rubber

PDMS base and curing agent were mixed at a mass ratio of 20:1, according to the literature [46] and stirred for five minutes, followed by addition of carbon black (4:1 mass of PDMS:CB). The resulting mixture was then stirred well for three minutes to obtain conductive silicone rubber. This silicone rubber was then coated onto a pre-made PET mold, which was coated with the suspension prepared in stage (1). It was then placed in a 70 °C convection oven to dry and cure. The film product was cut into 13 mm × 13 mm square (sheets) for dielectric tests.

**Figure 1:**
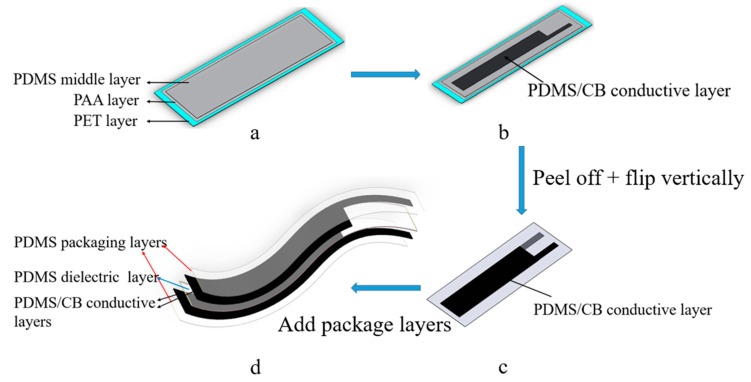
Schematic diagram of sensor fabrication process. (**a**) Low cost materials silicone (PDMS) coated on polyethylene terephthalate (PET) layer sprayed with poly(acrylic acid) (PAA). (**b**) PDMS/carbon black (CB) blend was coated on the PDMS dielectric layer’s surface. (**c**) Release of PDMS from PET surface with water, with PDMS/CB blend then coated on the other side of the PDMS dielectric layer. (**d**) PDMS was coated on both sides of the PDMS/CB blend layers for sealing and packaging.

(3) Flexible sensor production

(i) PDMS was prepared at 20:1 silicone elastomer base to curing agent mass ratio, stirred for 5 min and then degassed under vacuum. The AFA film applicator was used to coat it on the PAA coated PET surface made in (1), with the HAS set at a thickness of 0.2 mm. Then it was 70 °C heat cured for 0.5 h (Figure 1a).

(ii) Uncured flexible conductive silicone rubber was prepared using the same method of Flexible conductive silicone rubber (2). It was coated on the cured PDMS of step (i) using a PET mold at a thickness of 0.2 mm. Then placed in a 70 °C oven to dry and cure (Figure 1b)

(iii) PDMS released by washing with water and then peeling off the PET base. It was further washed with water three times and then dried.

(iv) The cured stack was flipped and another flexible conductive silicone rubber was prepared and placed on top of it as in step (ii). This is shown in Figure 1c. The stack was again cured.

(v) Another PDMS layer (0.2 mm thickness) was added to each side and cured as above, to package and sealing the sensor.

#### 2.2.2. Test and Characterization

Conductivity was measured with an S914 dielectric loss test device with AS2853 High Frequency Generator (Shanghai Radio Instrument Factory, Shanghai, China), at 10, 100 and 1000 kHz.

Capacitance was measured with TH2638 Capacitance Meter from Changzhou Tonghui Company (Changzhou, China). The measurement signal frequency was 1 kHz. Sampling rate was ~0.5 Hz unless otherwise specified. PDMS/CB electrodes were gripped by alligator clips for electrical connection. 

Surface morphology was characterized with an SU1510 scanning electron microscope (SEM) from Hitachi Company Japan (Tokyo, Japan).

Tensile tests were conducted on an electronic universal testing machine (SUST, Zhuhai, China) at an elongation rate of 5 mm min^−1^ and at room temperature (25 °C). The dimensions of each test sensor were 10 ± 0.1 mm × 3 ± 0.1 mm × 0.9 ± 0.05 mm. The span length was 40 mm.

**Figure A1:**
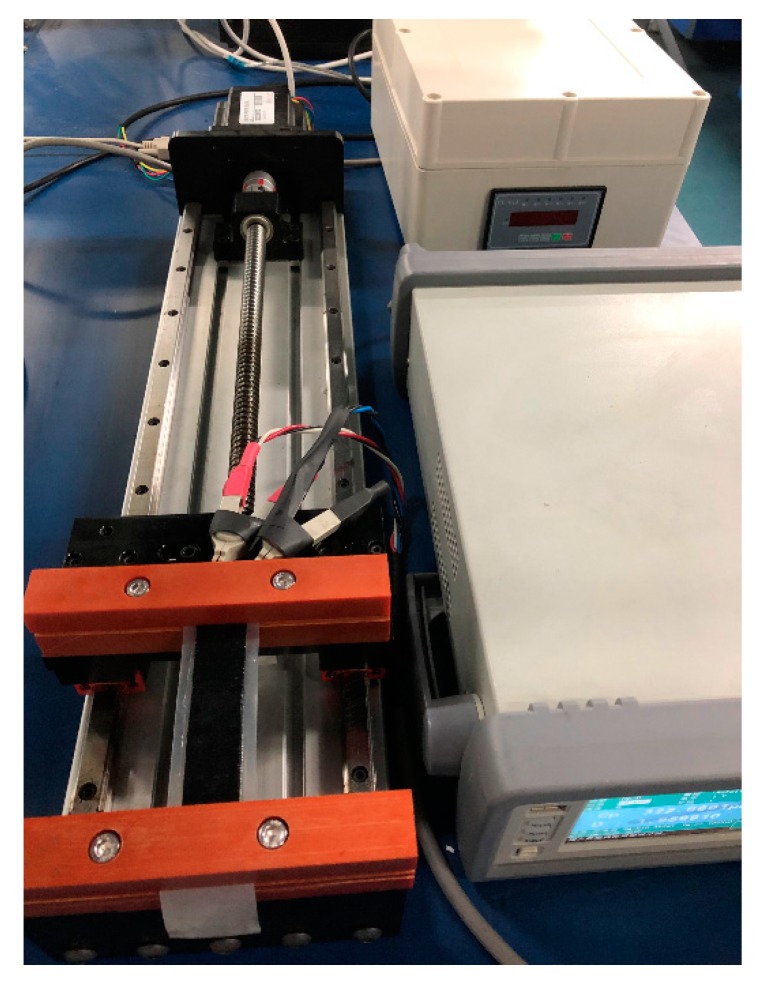
The reciprocating strain cycle tensile testing machine prepared by the investigators for durability testing. The flexible sensors are clamped at both ends to the machine. Required elongation and strain frequency are set with computer software, which controls a cyclic stepper motor.

Durability was tested by reciprocating strain cycling on a custom tensile test machine prepared in our laboratory (Figure A1). Straining frequencies of 0.1, 0.2 and 0.5 Hz were used, with elongations of 5%, 10%, 20% and 50%.

Further tensile tests were also conducted at different combinations of temperature and humidity. This was performed by enclosing the above custom tensile test machine in a custom chamber, where those parameters could be fixed. Temperatures of 15, 25 and 37 °C were combined with relative humidity (RH) values of 20, 40 and 60%. Strain rate was 0.5 Hz.

Monitoring of wearable applications was performed by fixing the sensors to paper elbow and knee bands with PDMS and measuring capacitance as described above. All monitoring was performed by the first author, with himself as subject. Elbow bending of up to 90° was recorded, with knee bending monitored during running and squatting and rising.

**Figure A2:**
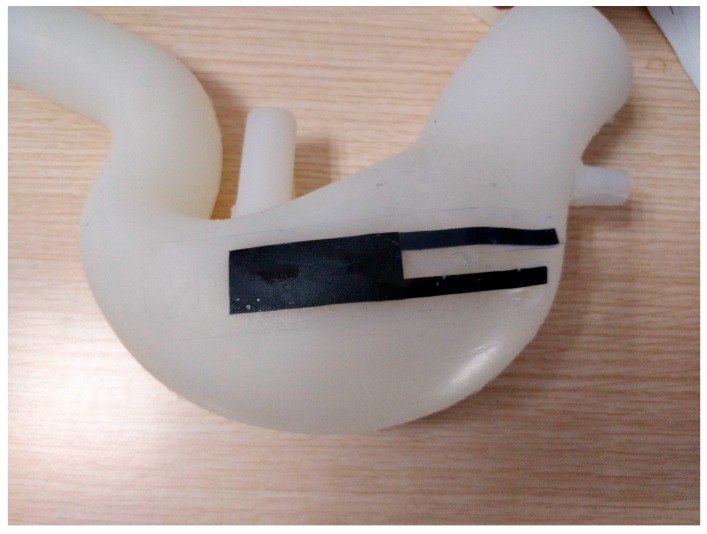
Placement of sensor on silicone in vitro stomach model—anterior wall of body.

Monitoring peristaltic deformation of the silicone stomach component of a human in vitro digestion model was achieved by laminating, with PDMS, a sensor to the anterior surface of its body, illustrated in Figure A2 of Appendix A. The stomach was compressed with rollers, simulating gastric motility. The model system was previously described by Wang et al. [54] A short video of the process is available in the Supplementary Material.

## 3. Results and Discussion

**Figure 2:**
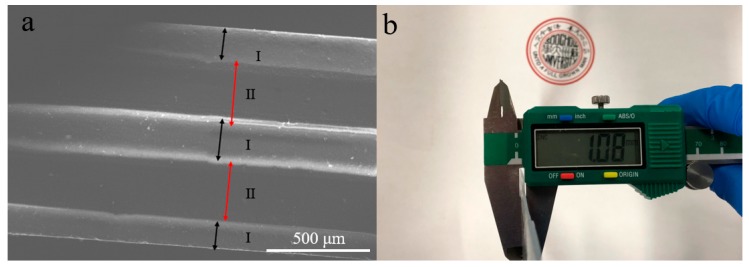
(**a**) SEM image of the flexible sensor at cross-section. The insulating layers are marked as I and the flexible conductive PDMS/CB layers marked as II. (**b**) Thickness of the assembled flexible sensor device. From top to bottom the individual layers were 175, 275, 200, 275 and 150 µm thick. Total thickness was 1.075 mm.

An approximately 1 mm thick, five-layer, flexible, PDMS based capacitive strain sensor was fabricated by sequential addition and curing of the layers. This process is low-cost and simple and is amenable to commercial scale-up via translation to roll-to-roll manufacturing, or other polymer film casting methods. In Figure 2a a cross sectional view SEM micrograph of the sensor is given, with the insulating layers marked as I and the flexible conductive silicone rubber (25% CB, see in Table A1) layers marked as II. The central insulator layer does not contain conductive carbon black and is the dielectric material and the outer two layers form insulating encapsulation, or packaging, layers for the sensor. The sensing mechanism is as follows: When the elastomeric sensor is stretched, the thickness of the dielectric layer decreases and the area of electrodes (A) increases, resulting in a significant increase in capacitance [22]. This is described by Equation (1). From the SEM micrograph, it can be seen that, from top to bottom, the individual layers were 175, 275, 200, 275 and 150 µm thick. Variations in the packaging layer thicknesses were due to variations in the bench-top fabrication method employed here. From Figure 2b, the overall thickness is 1.08 mm, consistent with the SEM image. The performance, fabrication method and characterization of comparable published flexible capacitive sensors are summarized in Table 1.

(1) $$C=\varepsilon_{0}\varepsilon_{r}\frac{A}{d}$$

where *C* is capacitance, *ε*_0_ and *ε_r_* are the dielectric constant of a vacuum and the relative permittivity of dielectric media, respectively, *A* is area of electrodes and *d* is thickness of the dielectric layer. 

Table A1 summarizes the conductive properties of different blends of PDMS and CB. It can be seen that as carbon content of the PDMS blends was increased from 15% to 30% electrical conductivity gradually increased, with useful conductivity achieved with 25% and 30% CB. Though it was the most conductive, the 30% CB silicone was difficult to prepare and exhibited rough surfaces, so 25% CB was used for sensor fabrication.

**Figure 3:**
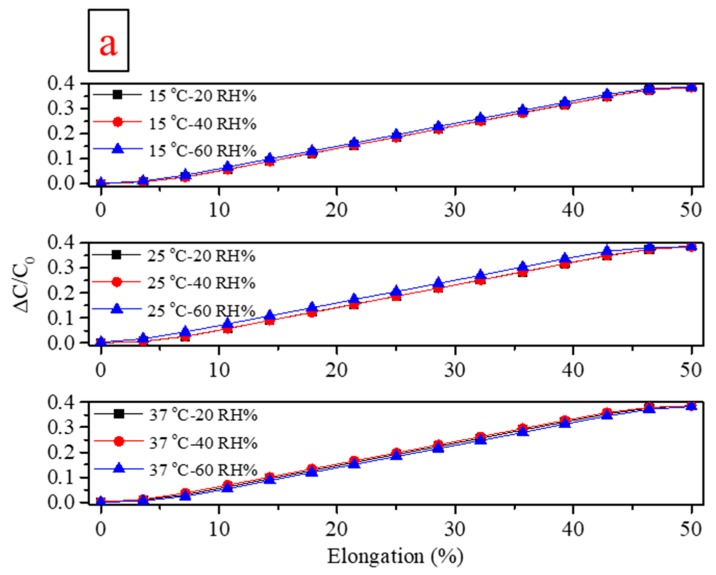

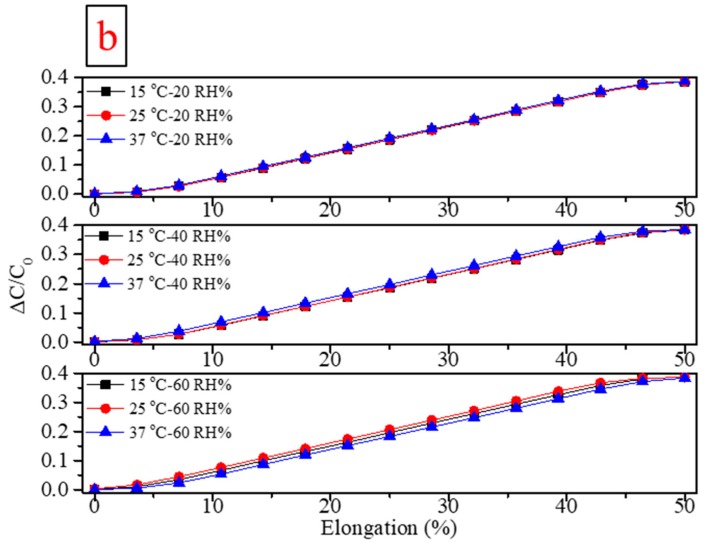
(**a**) Sensor capacitance measurements of 0 to 50% strain at constant temperature and varying humidity, recorded at 0.5 Hz strain rate. (**b**) Capacitance measurements at constant humidity and varying temperature, recorded at 0.5 Hz strain rate.

Figure 3a gives sensor ΔC/C_0_ values at fixed temperature [57] (15, 25, 37 °C) and varying relative humidity (20, 40, 60 %RH), over a range of 0 to 50 % elongation. Capacitance values measured at 25 °C and 40% RH ranged from ~260 to 380 pF and the total range of measurements was ~200 to 500 pF. These relatively large capacitive values are easily measured with miniaturized equipment and require much less sensitive data acquisition systems than some other [32] published sensors. For example, finger presses on smart phone capacitive touch screens typically register on the order of 1 pF change [58]. This also gives scope for device miniaturization.

It can be seen from Figure 3a that the ΔC/C_0_ varies little with humidity [59] when temperature is constant, with the most difference apparent at 25 °C. Conversely, Figure 3b gives Sensor ΔC/C_0_ values at fixed relative humidity (20, 40, 60 %RH) and varying temperature (15, 25, 37 °C). Again, little variance is displayed, with perhaps the most at 60 %RH. PDMS is water permeable [60] and will swell with water [61], but this effect was not apparent. The exterior PDMS packaging layers may have effectively isolated the internal conductive and dielectric layers, preventing significant influence from water vapor. Similarly, temperature has little influence over the measured range. Therefore, it can be concluded that temperature and humidity have at most only a minor effect on the sensor’s capacitive strain detection. These effects are unknown for most published sensor systems.

The sensor’s capacitive Gauge factor (G), defined by $\frac{\Delta C}{\Delta \varepsilon }\cdot \frac{1}{C_{0}}$, was also assessed at the different temperature and humidity combinations, with the values given in Table 2. It was very stable and within the median range for for published systems (~0.5 to 1.0). Table 1 makes this comparison with several comparable publications. 

**Figure 4:**
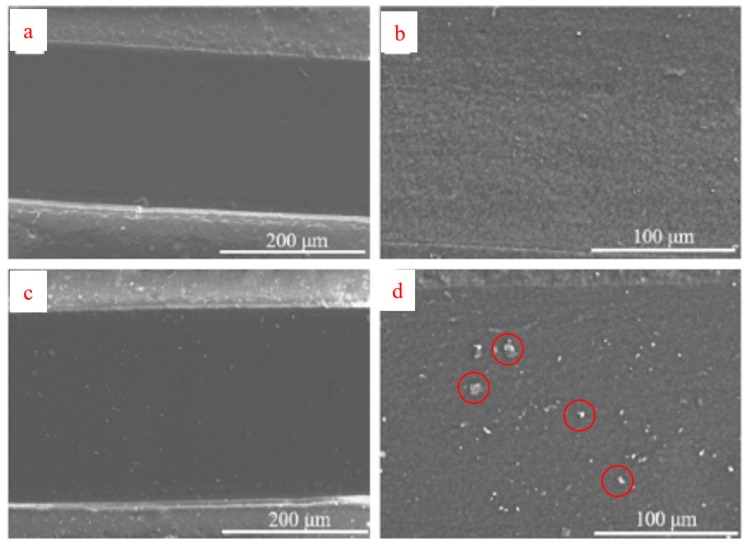

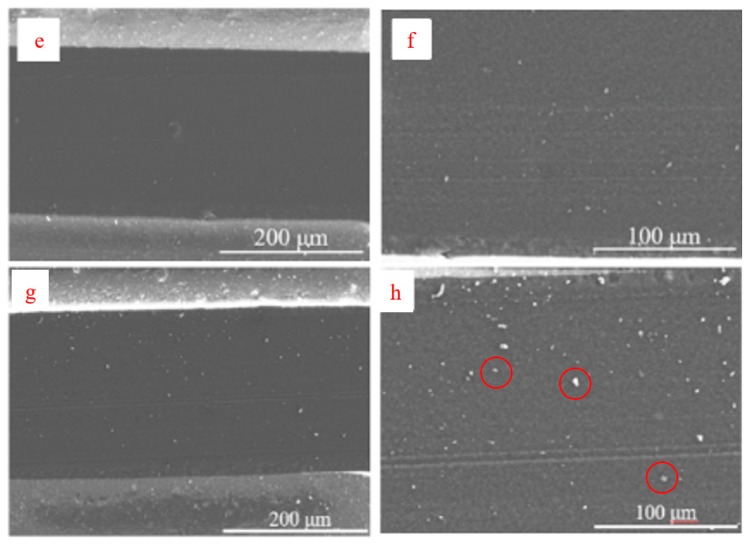
(**a**,**b**) SEM images, at different magnifications, of the flexible sensor’s conductive PDMS/CB blend layer at cross-section, before strain-cycling endurance test at 25 °C. (**c**,**d**) After 50% elongation strain-cycling endurance test at 25 °C. (**e**,**f**) Before strain-cycling endurance test at 37 °C. (**g**,**h**) After 50% elongation strain-cycling endurance test at 37 °C. Suspected carbon black particles are more visible in the higher magnification “after” images of (**d**,**h**). Red circles have highlighted selected particles to illustrate this.

Figure 4a,b is SEM cross-sectional views of the sensor taken before being subjected to 10,000 strain cycles. It can be seen from the figure that the surface of the sample is relatively flat before stretching. Figure 4c,d is SEM cross-sectional views of the sensor taken after endurance testing at 25 °C (50% strain). Small rough patches of ~10 μm size are infrequently observed. Figure 4g,h shows the SEM profile of the sensor taken after endurance testing at 37 °C (50% strain). Again small rough patches of ~10 μm size are sparsely distributed. These do not appear to be distinct cracks [47], but rather may be carbon particles disturbed by the strain-cycling, though it is not possible to be certain without further chemical imaging. Microcracks may have occurred that were not visible at this scale of magnification. Most importantly, sensor conductive layer morphology did not appear to change appreciably over 10,000 strain cycles of testing, demonstrating a seldom seen level of durability. 

**Figure 5:**
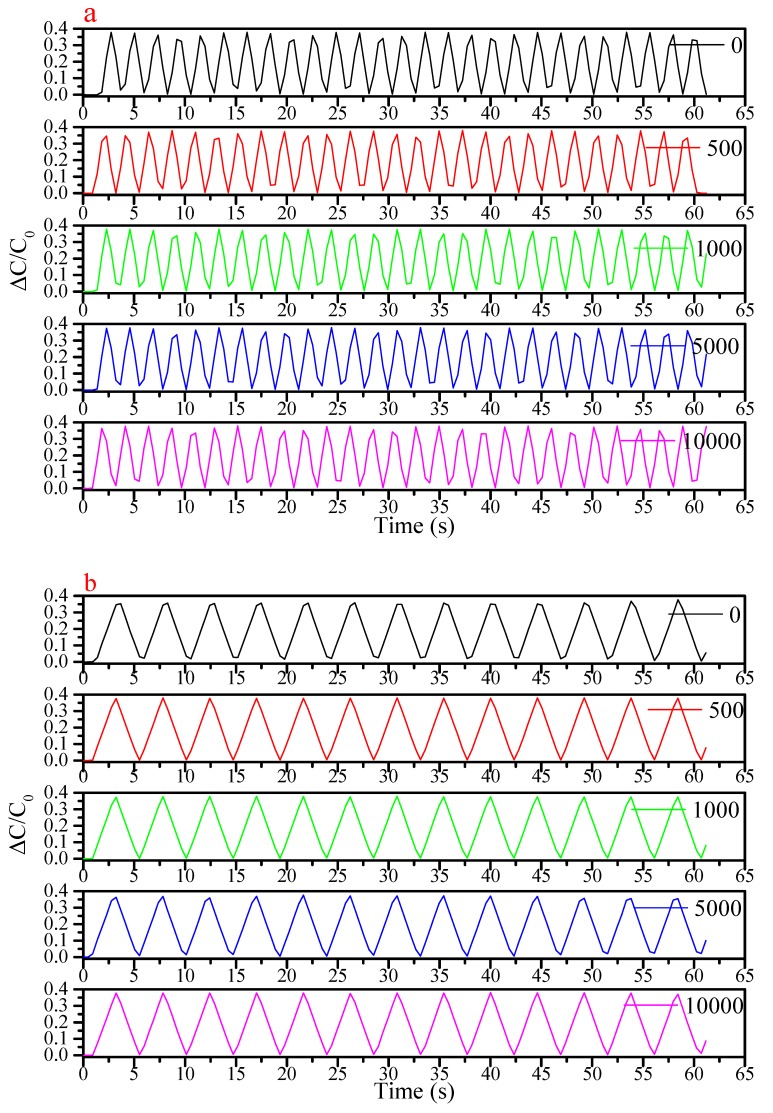

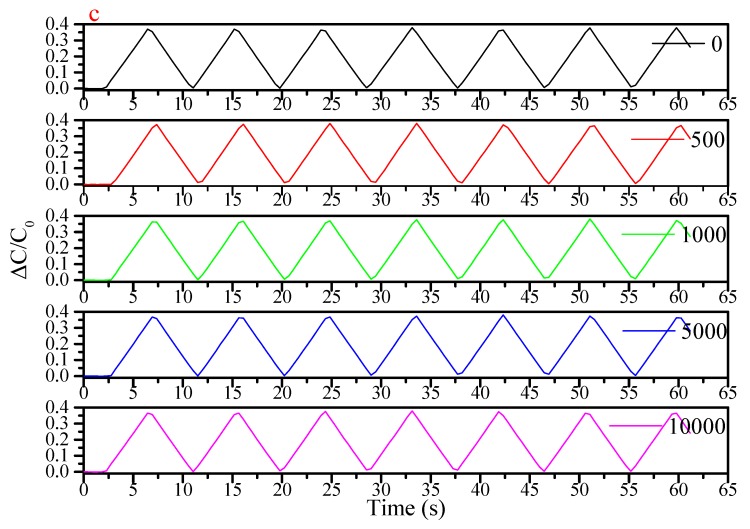
(**a**) Sensor record at the frequency of 0.5 Hz with 50% elongation at different times of the durability test. (**b**) At the frequency of 0.2 Hz with 50% elongation. (**c**) At the frequency of 0.1 Hz with 50% elongation.

Consistency of sensing performance with use was established by examining repeated strains at various elongations and frequencies, after 0 to 10,000 strain cycles. These results are illustrated in Figure 5, where consistent magnitudes of relative change in capacitance were observed. More variance within these strain cycle test groups was observed at the 0.5 Hz cycling frequency, but this may have been due to the limitations of the measurement, which restricted signal sampling to the same 0.5 Hz frequency.

**Figure A3:**
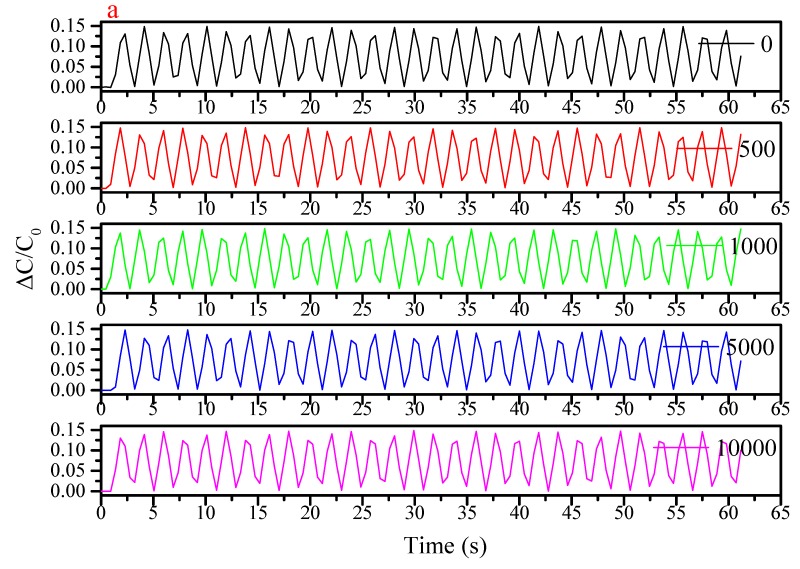

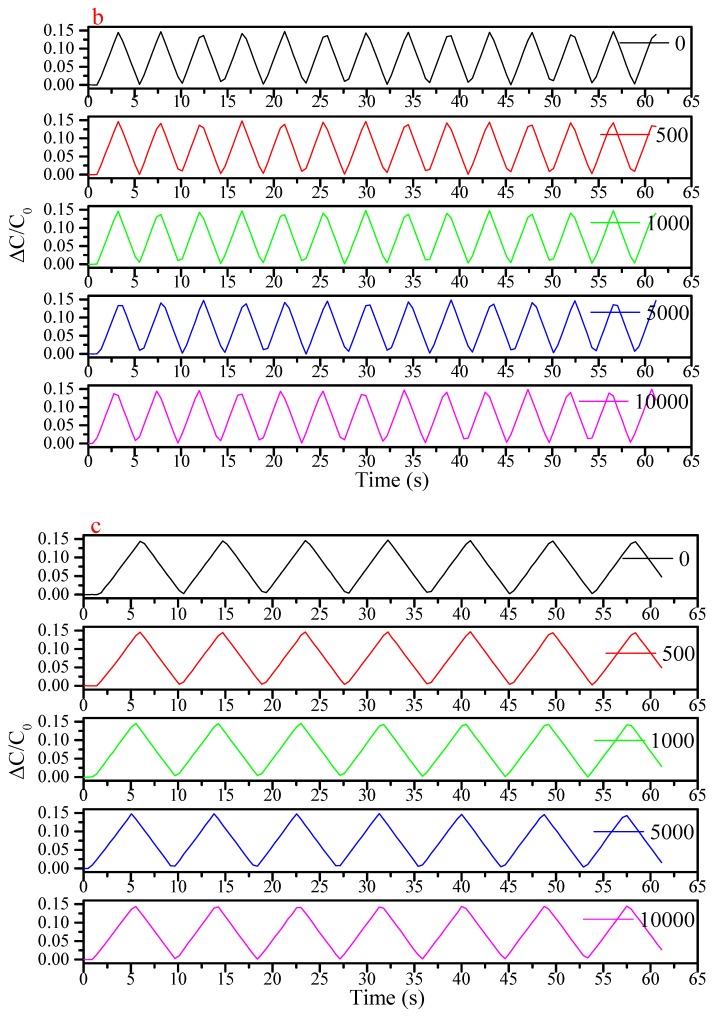
(**a**) Sensor record at the frequency of 0.5 Hz with 20% elongation at different times of the durability test; (**b**) at the frequency of 0.2 Hz with 20% elongation; (**c**) at the frequency of 0.1 Hz with 20% elongation.

**Figure A4:**
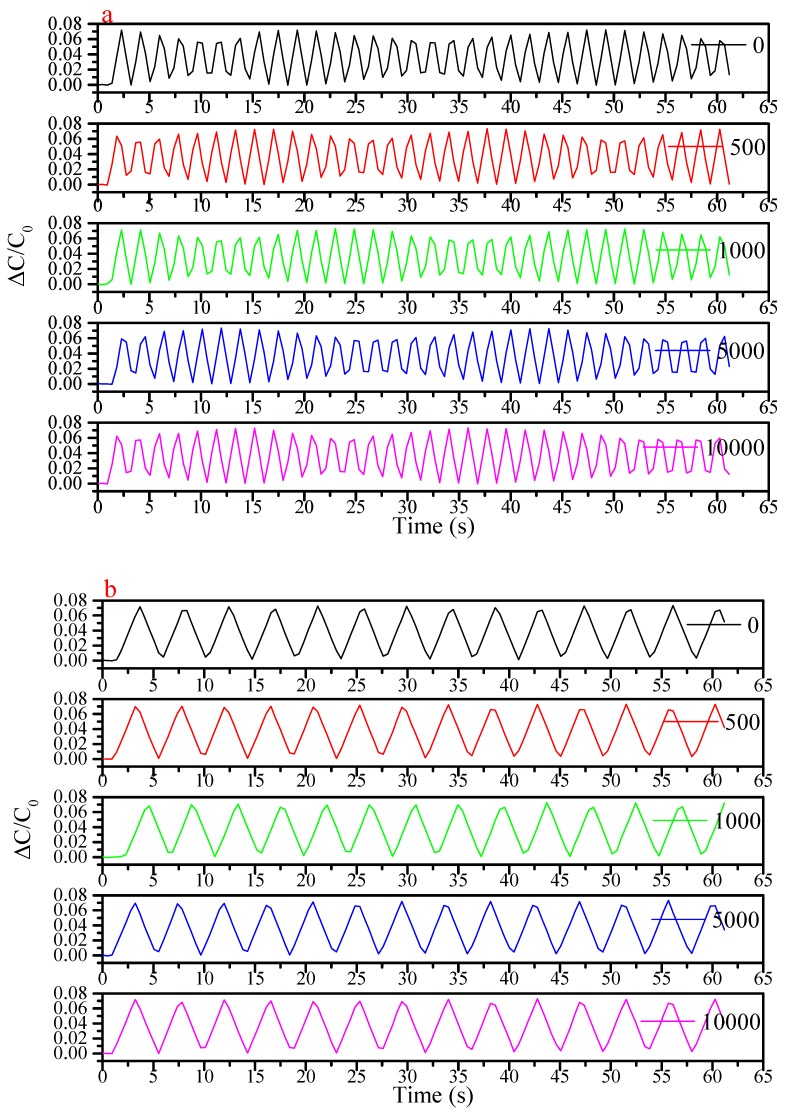

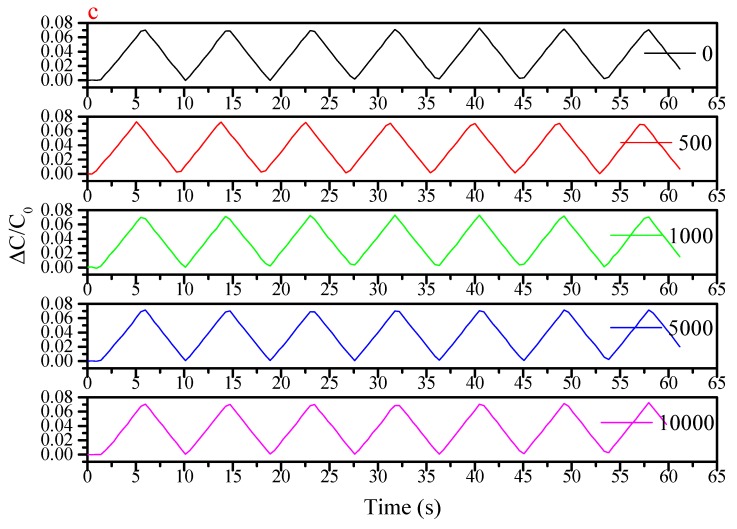
(**a**) Sensor record at the frequency of 0.5 Hz with 10% elongation at different times of the durability test; (**b**) at the frequency of 0.2 Hz with 10% elongation; (**c**) at the frequency of 0.1 Hz with 10% elongation.

**Figure A5:**
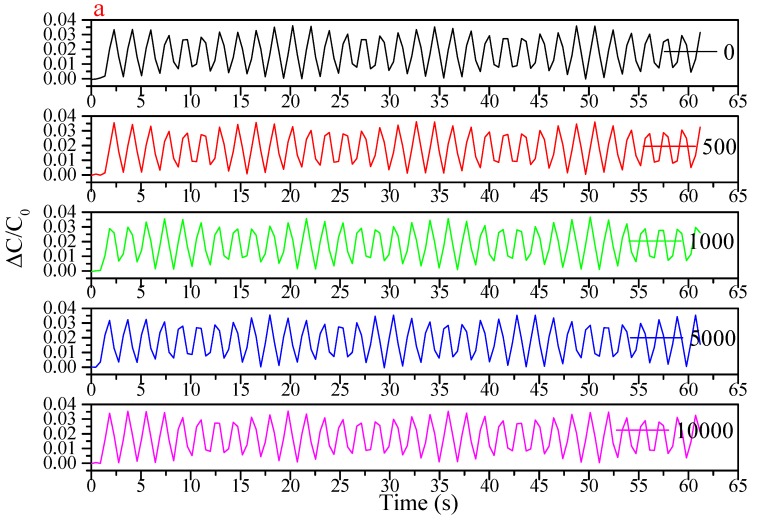

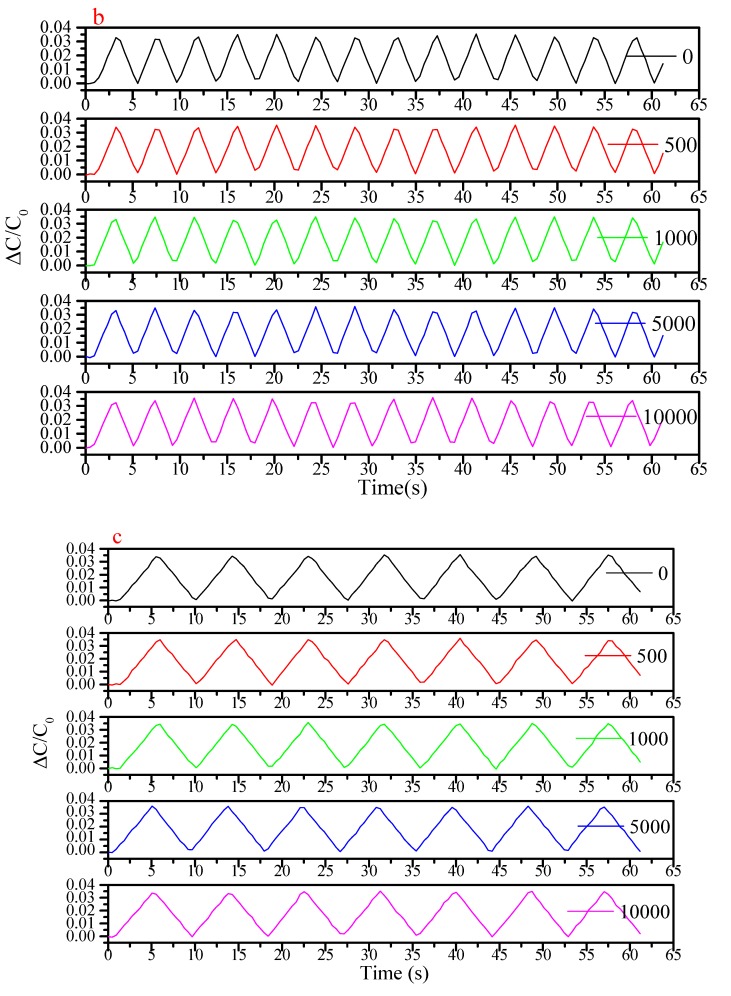
(**a**) Sensor record at the frequency of 0.5 Hz with 5% elongation at different times of the durability test; (**b**) at the frequency of 0.2 Hz with 5% elongation; (**c**) at the frequency of 0.1 Hz with 5% elongation.

It can be seen from Figure 5 that the sensor stretched at 0.5 Hz with 50% elongation for 100 times, 500 times, 1000 times, 5000 times and 10,000 times, separately, perform well. The value of the ΔC/C_0_ is 0.35. Even with different strain frequency, the high value of the ΔC/C_0_ is also 0.35. This consistency is observed again in Figure A2, Figure A3 and Figure A4. In Figure A3, with 20% elongation, the value of ΔC/C_0_ is consistently 0.14, which does not vary with strain frequency. This is also true of 10% elongation results of Figure A4 and the 5% elongation values of Figure A5, with ΔC/C_0_ is also 0.07 and 0.035 respectively. 

**Figure 6:**
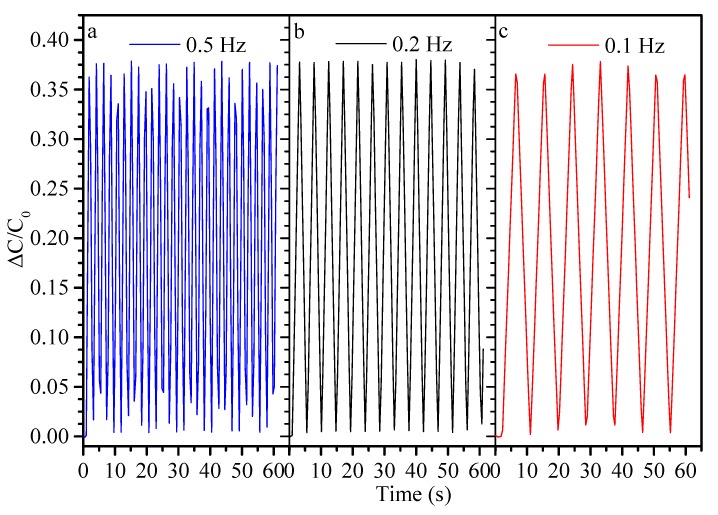
Relative capacitance changes at 50% elongation, for different strain cycle frequencies, after 10,000 strain cycles.

Finally, Figure 6 displays side-by-side repeated strain cycles, recorded at different frequencies after 10,000 cycles, for 50% elongation. Again stability of signal and durability, are displayed by the modest variance in capacitive change. This confirms the results of Shintake et al. [53] with a similar 5-layer PDMS/CB sensor, who earlier demonstrated durability over 10,100 cycles. These are the only known studies that have examined durability to this extent and have demonstrated wearable applications of their sensor materials.

Cohen et al. [20] also demonstrated signal stability with their CNT based capacitive strain sensor, but over 3000 strain cycles, while He et al. [27] showed similar signal stability for their graphene based capacitive pressure sensor over 1050 cycles of strain. The greatest duration study of capacitive sensor stability was performed by White et al. [56], who examined an IGT/silicone blend and silicone based multi-layer sensor over 100,000 strain cycles, observing considerable performance deterioration after 25 k cycles. This indicates that even further durability testing may need to be performed in future.

**Figure 7:**
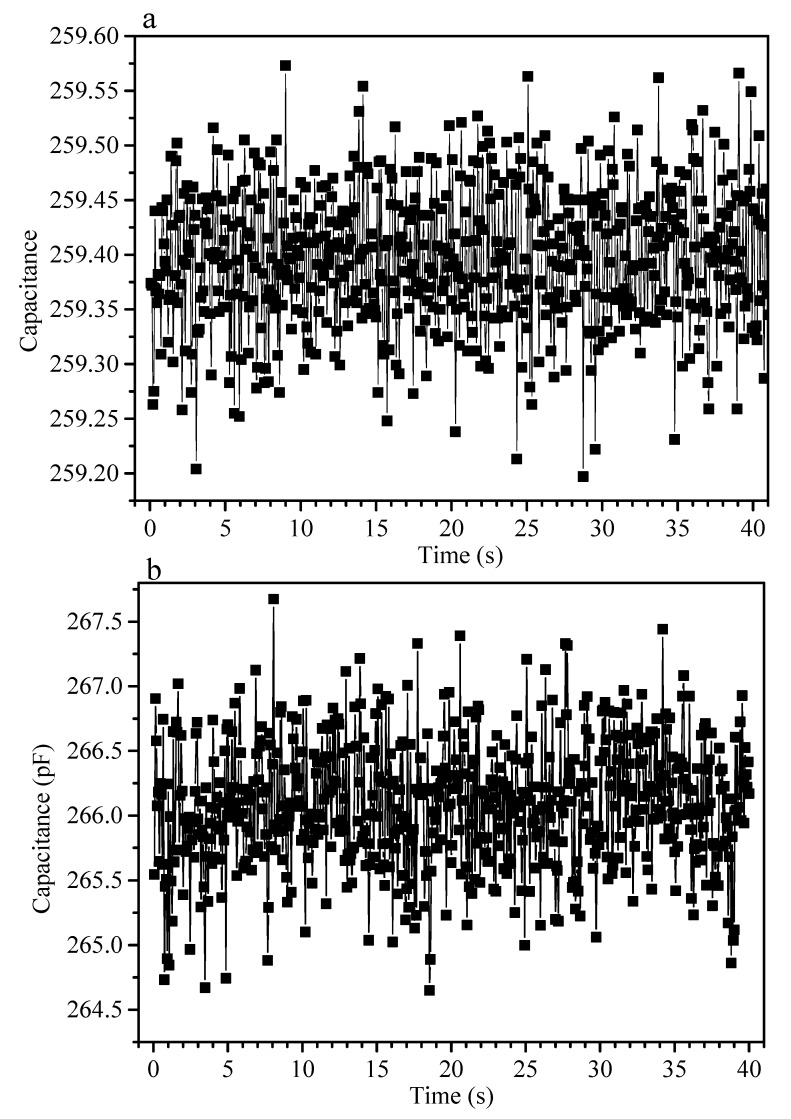
(**a**) Capacitance signals measured from a wearable sensor at rest on table. (**b**) Signals obtained when the wearable sensor is attached to the skin of a stationary human elbow. Measurements were made at 2.5 Hz sampling frequency.

Figure 7 displays the measured capacitance of a wearable version of the sensor, when it is at rest on a table and when it worn on the stationary elbow of a human subject, where it is in contact with the skin. Wearability was achieved by integrating the sensor with an elbow band. Mean capacitance was 259.4 ± 0.2 pF on the bench, where the standard deviation is equivalent to ΔC/C_0_ of ±0.0008. Mean capacitance was 266 ± 1.5 pF on the stationary elbow, where the standard deviation is equivalent to ΔC/C_0_ of ±0.0056. Attaching the sensor to skin had the effect of a one off capacitance increase of ~2.5%, easily compensated for. Increased signal variance from skin contact had little impact [62,63] on the measurement result.

**Figure 8:**
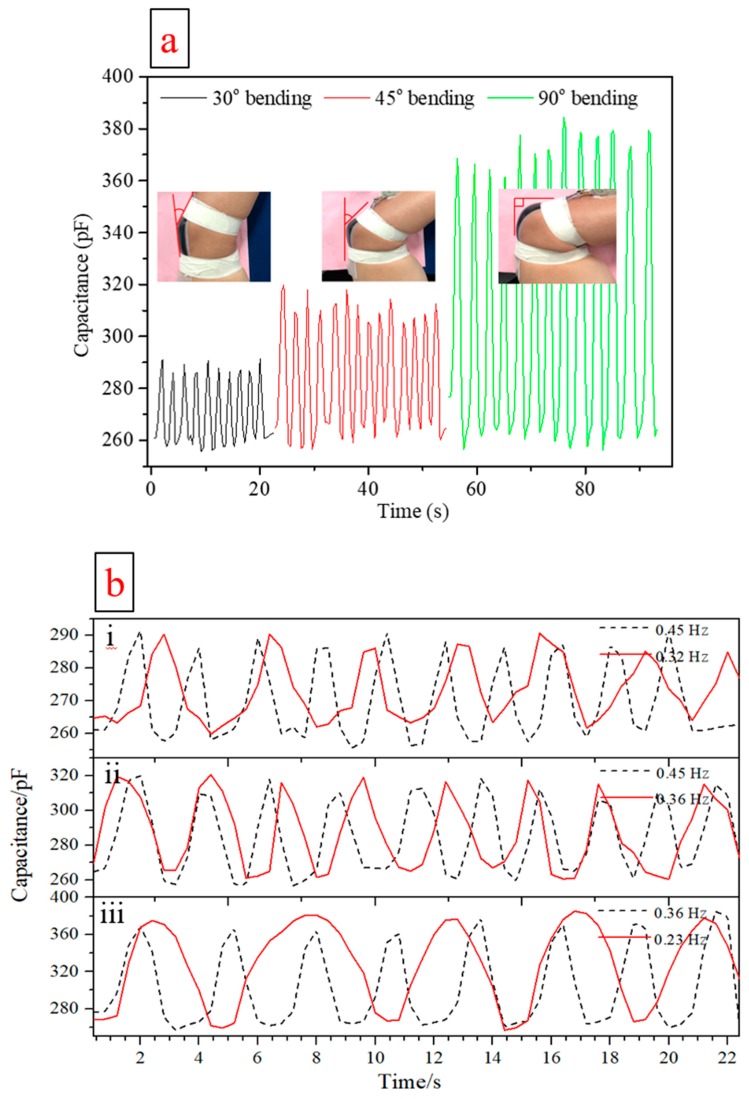
(**a**) Typical capacitance changes recorded with the flexible sensor when monitoring human elbow motion over different flexion angles. (**b**) Sensor capacitance signals recorded during elbow flexion at different motion frequencies; (b-i) for 30° elbow bending; (b-ii) for 45° elbow bending; (b-iii) for 90° elbow bending.

Figure 8a illustrates monitoring of elbow flexion angles of 30°, 45° and 90°, during repetitive motion. The capacitance varies from ~260 to 285 pF for bending from 0–30°, from ~260 to 310 pF for bending from 0–45° and from 260 to 375 pF for bending from 0–90°. As the flexion angle increases, the maximum value of the capacitance increases accordingly. As the bending angle becomes larger, sensor area increases and the spacing between the two flexible electrodes becomes smaller. This relationship is in keeping with the capacitance formula C = ε_0_ε_r_ (A/d) [44], where ε_0_ is the space permittivity, ε_r_ is the relative dielectric constant of the dielectric material, A is the area of the capacitor and d is the distance between separated electrodes. The sensor appears to report fairly repeatable and stable signals during testing at different flexion angles, with much of the variance likely due to varying degrees of motion from the human test subject, or the 0.5 Hz sampling rate limit of the data acquisition system. Relative signal changes, of ~40% over the full range of motion, given by ΔC/C_0_, give clear indications of this motion and due to the 0.86 gauge factor, are comparable to the highest range observed in this field [64].

The results of testing of the sensor at different elbow flexion angles and at two different repetitive movement frequencies for each flexion angle are shown in Figure 8b. It can be seen that the characteristics of these under these different motions can be identified well, reflecting the movements of the human arm. In addition, the characteristics are generally repeatable for the same maximum bending angle and the magnitude of capacitance change increases with increasing flexion angle. All of the above suggest that the current sensor, despite its simplicity, functions well for large angle bodily motion detection.

**Figure 9:**
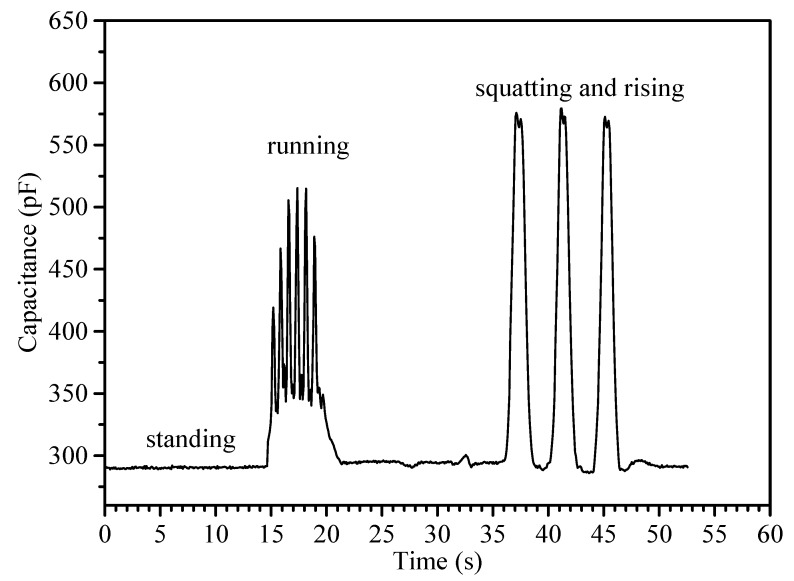
Typical capacitance changes measured with the flexible sensor while monitoring human knee motion over different large flexion angle movements.

Further characterization of the device for large flexion angle motion was achieved via fixing it to knee band and monitoring capacitance during running and squatting and rising. This is illustrated in Figure 9. The motions are clearly distinguished, with relative signal changes up to ~90%, again comparable with the highest range observed in this field. [64] Together, these results represent the first application of a CB/silicone based capacitive strain sensor to this class of motion.

**Figure 10:**
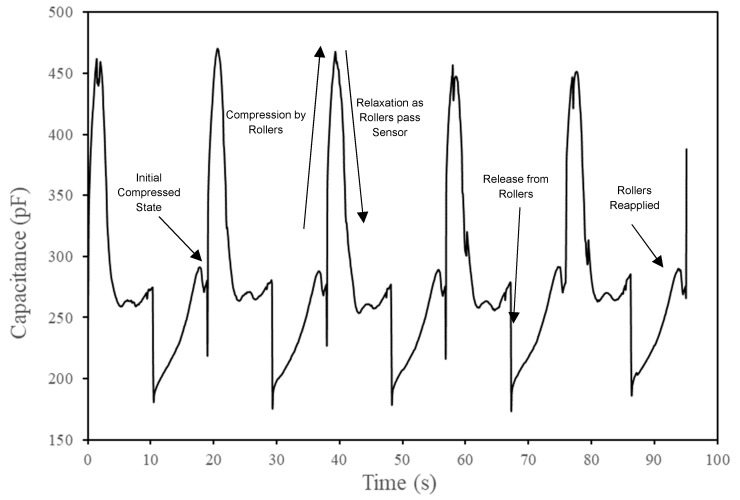
Capacitive signal trace from monitoring silicone in vitro stomach model peristaltic compression, simulating gastric motility. Labels indicate the different phases of motion and signal detection.

A final application of the sensor material was used to gauge its suitability for monitoring large scale soft tissue deformation, of the type that would be seen with mammalian organs. This was achieved by laminating the sensor to an anatomically realistic, silicone stomach model, from an in vitro human digestion simulation system [54]. Sensor position is illustrated in Figure A2 from Appendix A and is located on the anterior body wall. The peristaltic motion that drives gastric motility is simulated by applying rollers to the model and a brief video of this process is given in the Supplementary Material. The stomach is initially compressed in the default position, which increases as the rollers move, eventually moving past the model and allowing it to relax. The rollers then return to a position at the other end of the model, where they change direction and resume the original motion. The compression and relaxation phases of this process are easily distinguished in Figure 10, a capacitive signal trace of several iterations. A video of the process has been included in the Supplementary Material.

This experiment further demonstrates the suitability of the sensor material to monitoring large scale human motions, this time through compression deformation of soft tissue and may be the first such application to this type of motion. Currently this study has been limited to in vitro models, which indicate that the material can be used in these systems, or externally on the body. Further development may include surface modification for use in animal models of disease. 

## 4. Conclusions

A flexible capacitive strain sensor was developed from the low-cost materials silicone and carbon black and effective characterization for large flexion-angle motion and large tissue deformation applications of such a device was performed. The fabrication process was simple and amenable to commercial scale-up, with successive silicone layers cast and cured such that blended PDMS/CB electrodes entrapped a PDMS dielectric layer, with these three elements then packaged and sealed by exterior PDMS films. Sensing performance was characterized by the capacitive gauge factor, which was measured at 0.86 over 3 to 47% strain, comparable with the median range found in flexible sensors (~0.5 to 1.0). This performance showed almost no variation over temperature and relative humidity ranges of 15 to 37 °C and 20 to 60% and nor did relative capacitance. Durability was established by subjecting sensors to 10,000 strain cycles, with varying strain frequency and elongation up to 50%. Consistent relative capacitance changes were obtained across the full range of this testing. 

Suitability for large flexion-angle human motions was demonstrated by integration of the prepared sensor with elbow and knee bands, where it allowed detection of high flexion angle (90°) human motion, with large changes in relative capacitance measured (~40% to 90%). Large tissue deformation was monitored by integration with an anatomically realistic, silicone stomach model, from an in vitro human digestion simulation system. Simulated gastric motility was clearly observed.

This device realizes the promise of low-cost silicone and carbon black materials for capacitive strain detection of large scale human motion, with a robust device that is amenable to commercial scale production and has been characterized for wearable use. Future development may include miniaturization, surface modification for use in animal models of disease and further characterization in wearable applications—its mechanical durability, simple construction and electrical signal range may be attractive for athletic performance monitoring. 

## Figures and Tables

**Table 1:** Summary of performance of flexible capacitive strain and pressure sensors reported in the literature. Sensors were evaluated over four aspects: Fabrication process, capacitive gauge factor (G) for strain sensors, characterization as a wearable sensor and characterization of sensor durability. This sensor’s G factor was within the median of the reported range, though < 1. Of the strain sensors characterized as wearable devices only Shintake et al. [53] and this sensor were evaluated for durability (10 k cycles).

Flexible Conductive Material	Ref.	Flexible Conductive Layer Manufacturing Process	Sensor Type	G Factor	Wearable Experiment	Durability Testing
CNT	[20]	CNT stamping on patterned plasma treated silicone	Capacitive strain	0.99	×	3000 cycles
	[23]	CNT sprayed on UV/Ozone activated PDMS layers via patterned mask, PDMS layers fixed with silicone adhesive	Capacitive strain	0.4	×	×
	[25]	Fluorinated substrate, sprayed w/ CNT through mask, PDMS cast and cured on substrate to transfer CNT	Capacitive strain	0.41, 0.68 (x, y dir)	×	×
Graphene	[26]	Graphene oxide (GO) foam between reduced GO (rGO) patterned PET substrates	Capacitive pressure	-	×	1000 cycles
	[27]	CVD deposited graphene transferred to PMMA then to PDMS, nylon mesh and silver electrode sandwiched between two graphene-PDMS layers	Capacitive pressure	-	√	1050 cycles
	[28]	AgNW and rGO spin-coated on patterned, plasma treated PDMS, then polyurethane (PU) coated. Two composites fixed together to form device	Capacitive pressure	-	√	×
Carbon Black	[29]	Screen printing of silicone and silver-silicone adhesive electrode, layers, with blended silicone/CB dielectric; silicone adhesive layers used	Capacitive pressure	-	√	×
	[30]	Curing and gluing together successive blended PDMS/CB electrode layers and PDMS layers (dielectric, packaging)	Capacitive strain	1	×	×
	[53]	Casting and curing of successive Ecoflex silicone layers (dielectric, packaging), with blended silicone/CB electrode layers encapsulated by silicone layers. Devices cut from material by laser	Capacitive strain	0.83 to 0.98	√	10,100
	This work	Casting and curing of successive PDMS layers (dielectric, packaging), with blended PDMS/CB electrode layers	Capacitive strain	0.86	√	10,000 cycles
Expanded Intercalated Graphite [IGT]	[56]	Expanded graphite blended with silicone—two layers encapsulated silicone dieletric layer. Sensors film cast, screen printed, or 3D printed	Capacitive strain	0.54 to 1.13	x	100,000 cycles
Nanowires	[21]	AgNW cast and patterned on Si wafer, transferred to PDMS by casting and curing, bonded to second PDMS-AgNW layer	Capacitive strain	1	×	×
	[22]	AgNW pattern on Si wafer by screen printing, transferred to PDMS by casting and curing, Cu wire and liquid metal sandwiched between two AgNW-PDMS layers, secured with Ecoflex	Capacitive strain	0.7	√	×
	[31]	AgNW cast on glass, transferred to PU by casting and curing. Two composites laminated with acrylic dielectric spacer inside	Capacitive strain	0.5	×	×

**Table 2:** Sensor capacitive gauge factor (G) and linearity at different temperature and humidity, assessed over the linear range (~3 to 47% strain).

Test Conditions	R^2^	Gauge Factor
15 °C–20 RH%	0.9945	0.86
15 °C–40 RH%	0.9947	0.86
15 °C–60 RH%	0.9950	0.86
25 °C–20 RH%	0.9947	0.86
25 °C–40 RH%	0.9944	0.86
25 °C–60 RH%	0.9931	0.85
37 °C–20 RH%	0.9950	0.86
37 °C–40 RH%	0.9947	0.86
37 °C–60 RH%	0.9942	0.85

## Supplementary Materials

The following are available online at https://www.mdpi.com/1424-8220/19/20/4553/s1.

## Appendix A

**Table A1:** Effect of carbon black addition on PDMS conductivity.

Sample	(m_PDMS_:m_Carbon_)	Surface Roughness	Conductivity (S/cm)		
			10 kHz	100 kHz	1 MHz
S1	100:15	smooth	7.28 × 10^−7^	4.49 × 10^−6^	2.71 × 10^−5^
S2	100:20	smooth	5.35 × 10^−6^	2.15 × 10^−5^	1.83 × 10^−4^
S3	100:25	intermediate	6.58 × 10^−5^	8.34 × 10^−5^	3.04 × 10^−4^
S4	100:30	rough	9.05 × 10^−4^	1.01 × 10^−3^	1.42 × 10^−3^

From Table A1, it can be seen that as carbon content of the PDMS blends was increased from 15% to 30% electrical conductivity gradually increased, with useful conductivity achieved with 25% and 30% CB. Though it was the most conductive, the 30% CB silicone was difficult to prepare and exhibited rough surfaces, so 25% CB was used for sensor fabrication.
